# Small Bowel Volvulus Secondary to Meckel’s Diverticulum With Meso-Diverticular Band: A Case Report and Literature Review

**DOI:** 10.7759/cureus.83817

**Published:** 2025-05-09

**Authors:** Solomon Raj Vasudayan, Guo Hou Loo, Mogaraj Sellapan, Geok Chin Tan, Nik Ritza Kosai

**Affiliations:** 1 Upper Gastrointestinal and Metabolic Surgery Unit, Department of Surgery, Faculty of Medicine, The National University of Malaysia, Kuala Lumpur, MYS; 2 Department of Pathology, Faculty of Medicine, The National University of Malaysia, Kuala Lumpur, MYS

**Keywords:** closed loop obstruction, congenital anomalies, meckel´s diverticulum, meso-diverticular band, small bowel volvulus

## Abstract

Small bowel volvulus (SBV) is a rare yet potentially fatal cause of intestinal obstruction characterized by the twisting of the small intestine around its mesenteric axis. Prompt diagnosis and timely intervention are critical to preventing complications such as bowel ischemia, perforation, and sepsis. SBV can occur without a clear underlying pathology (idiopathic) or be associated with anatomical anomalies, such as Meckel’s diverticulum (MD), accompanied by a meso-diverticular band (MDB). We report a rare case of SBV in an adult woman, resulting from MD complicated by the presence of an MDB. She presented with a two-day history of severe central abdominal pain, distension, vomiting, and obstipation. Imaging revealed features consistent with SBV without definitive identification of the underlying cause preoperatively. Urgent exploratory laparotomy was performed, identifying MD with MDB as the causative factors. The patient underwent small bowel resection and excision of MD along with the MDB. Surgical intervention remains the cornerstone of management for SBV, with early operative treatment crucial to minimizing morbidity and mortality. Although traditional laparotomy remains common, laparoscopic approaches are emerging as effective alternatives, offering potential advantages in diagnosis and management. This case underscores the importance of considering MD with MDB in adults presenting with SBV and highlights key surgical management strategies.

## Introduction

Small bowel volvulus (SBV) is a rare and life-threatening condition where the small intestine twists around its own mesenteric axis that results in bowel obstruction [1]. In the adult population, limited studies suggest an annual incidence of 1.7-5.7 per 100,000 adults in Western countries to 24-60 per 100,000 adults in Asian countries [1]. SBV can arise idiopathically, without any identifiable anatomical cause, or may result from underlying pathological conditions, including adhesion bands, neoplasms, and Meckel’s diverticulum (MD) [2]. While MD is often asymptomatic, it is usually discovered as a complication, namely gastrointestinal bleed, diverticular perforation, Meckel’s diverticulitis, and SBV [3]. SBV is a recognized complication of MD, occurring in approximately 5.5% of cases [3]. Despite its infrequent occurrence, SBV warrants vigilant attention and prompt management, as this condition may precipitate severe sequelae such as bowel ischemia, perforation, and sepsis [2]. We present an unusual case of SBV in an adult lady, secondary to MD with the presence of meso-diverticular band (MDB), which was attached to the anterior abdominal wall, causing a closed-loop obstruction. This case has been reported as per CARE guidelines [4].

## Case presentation

A 19-year-old girl presented with complaints of sudden-onset severe abdominal pain that initially started over the epigastric region, then progressed to the central abdominal region for the past two days. Described as colicky in nature, the pain was mildly alleviated by lying supine. Besides the pain, she had abdominal distension and vomiting episodes more than 10 times a day. The vomit content was mainly food particles, non-bilious, and non-feculent. She had obstipation that developed at the same time with the rest of the symptoms. Upon examination, she was not septic, and vitals signs were stable. Examination of the abdomen showed a grossly distended abdomen with tenderness over the epigastric and umbilical region; otherwise, she had no previous abdominal scars, not in peritonism, and hernial orifices were intact. Per rectal examination was unremarkable.

Initial blood investigations showed a raised white cell count of 13.0 x 10^9^/L with neutrophilic predominance (10.5 x 10^9^/L, 80.2%). Her hemoglobin was 13.8 g/dL, and platelets were elevated at 297 x 10^9^/L. Electrolytes and renal profile were within normal limits: sodium 136 mmol/L, potassium 4.3 mmol/L, urea 4.8 mmol/L, creatinine 66.4 umol/L. Serum lactate was mildly elevated at 1.2 mmol/L (normal range 0.5-2.2 mmol/L), suggesting a degree of early hypoperfusion without established ischemia.

**Figure 1:**
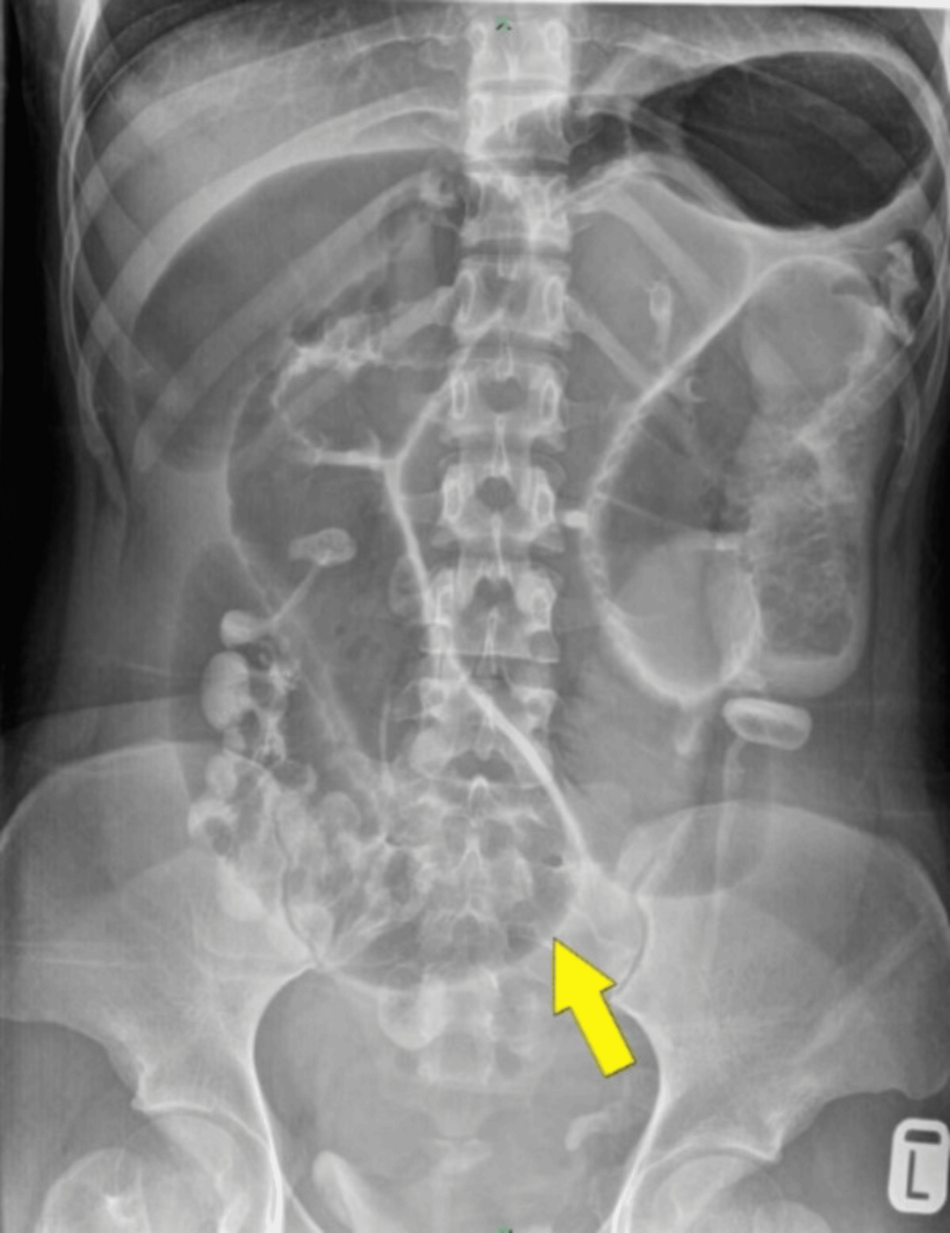
Plain abdominal X-ray in supine position showing grossly dilated small bowel loops (yellow arrow).

Abdominal X-ray showed centrally dilated small bowel (Figure 1).

**Figure 2:**
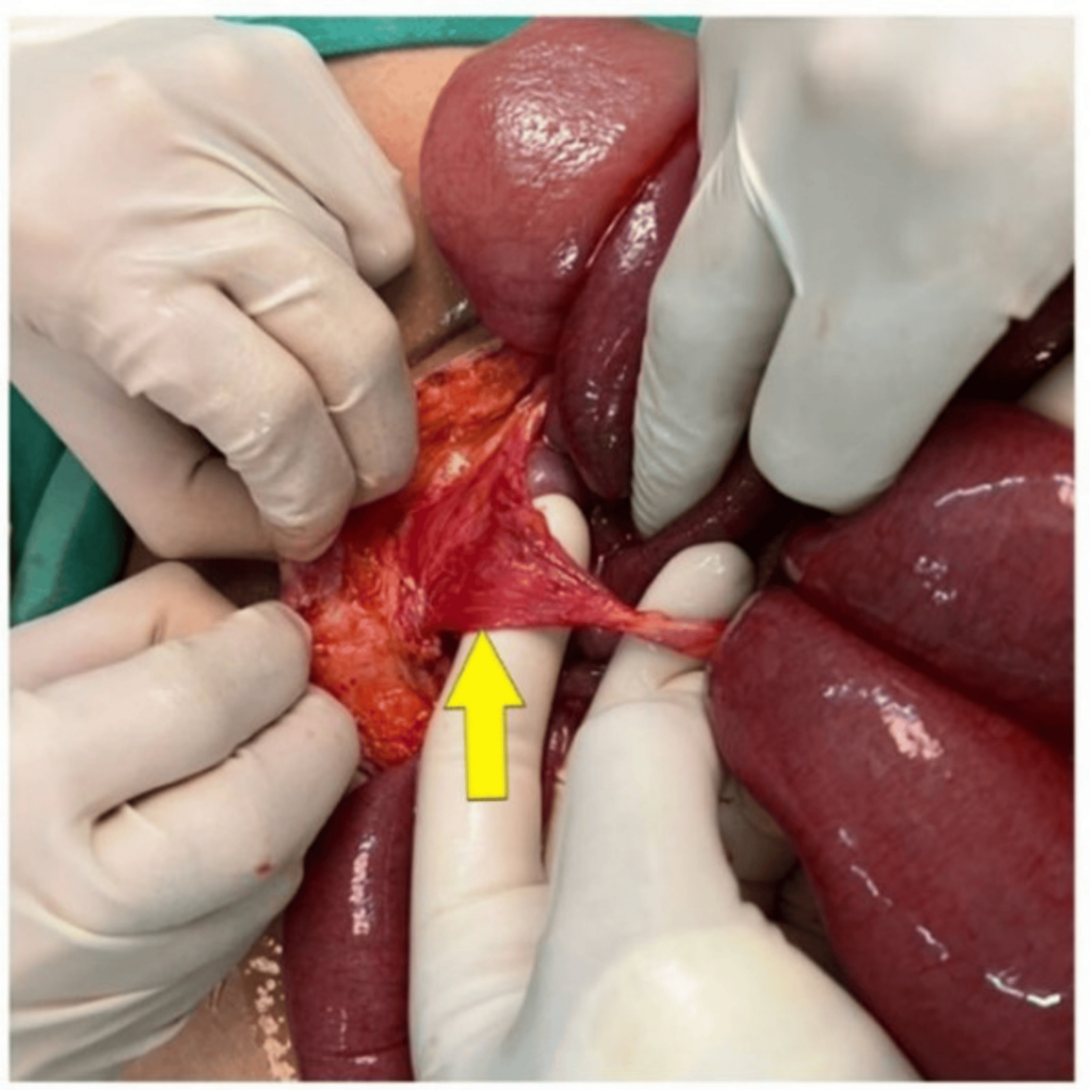
Intraoperative image showing meso-diverticular band attached to the anterior abdominal wall (yellow arrow) with grossly dilated small bowels.

**Figure 3:**
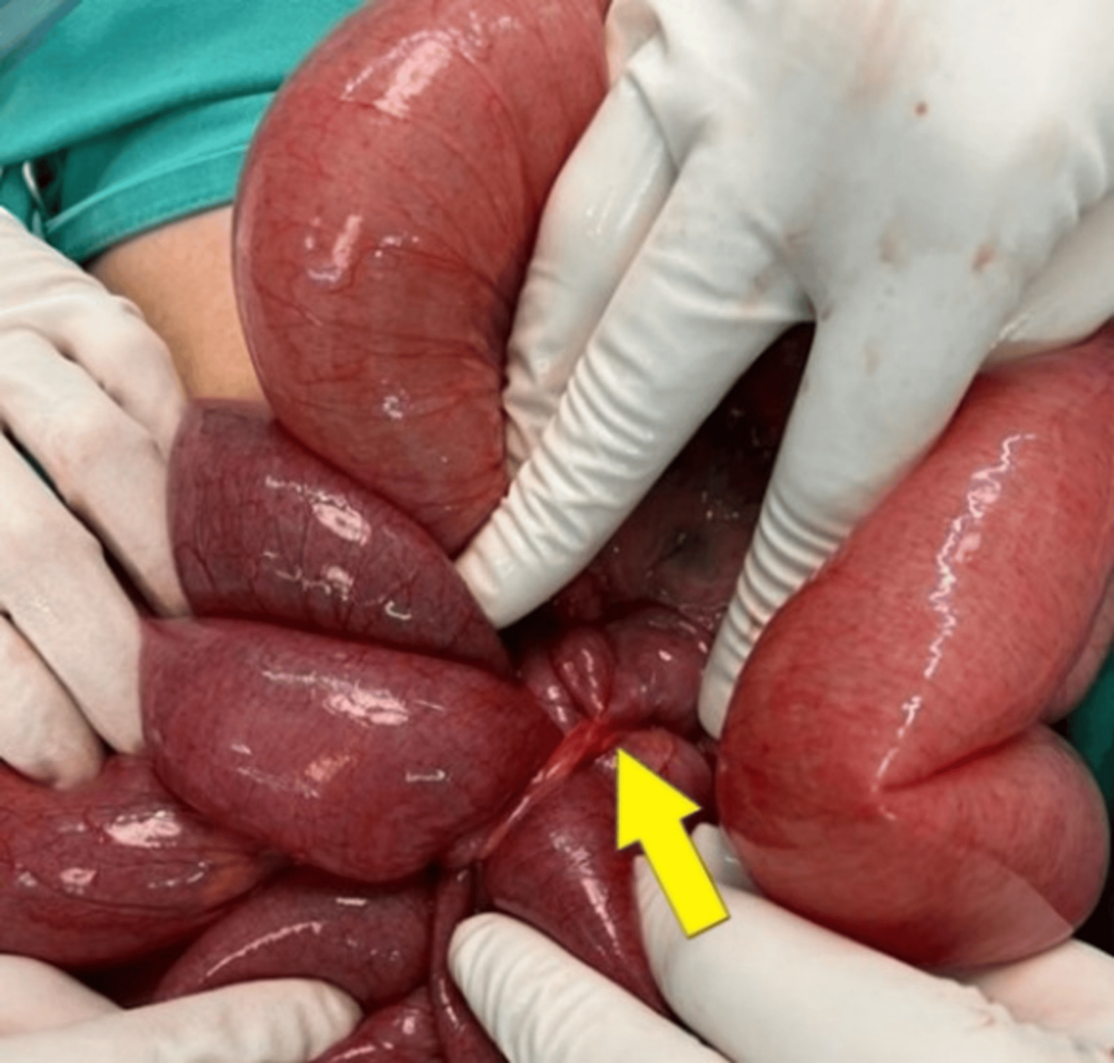
Intraoperative image showing meso-diverticular band in continuum with Meckel's diverticulum (yellow arrow) constricting the small bowel underneath, which results in a closed-loop obstruction.

A contrast-enhanced computed tomography (CECT) scan of the abdomen, performed at a private center prior to her presentation, revealed features of small bowel obstruction with possible volvulus involving the distal jejunum. As the underlying cause of the volvulus could not be clearly identified and given the urgent need to relieve the obstruction and prevent bowel perforation or further clinical deterioration, the patient was promptly resuscitated and proceeded to exploratory laparotomy. Intraoperatively, SBV was noted secondary to MDB attached to the anterior abdominal wall (Figure 2) with MD over the mesenteric border of the ileum 60 cm from the ileocecal junction (Figure 3).

**Figure 4:**
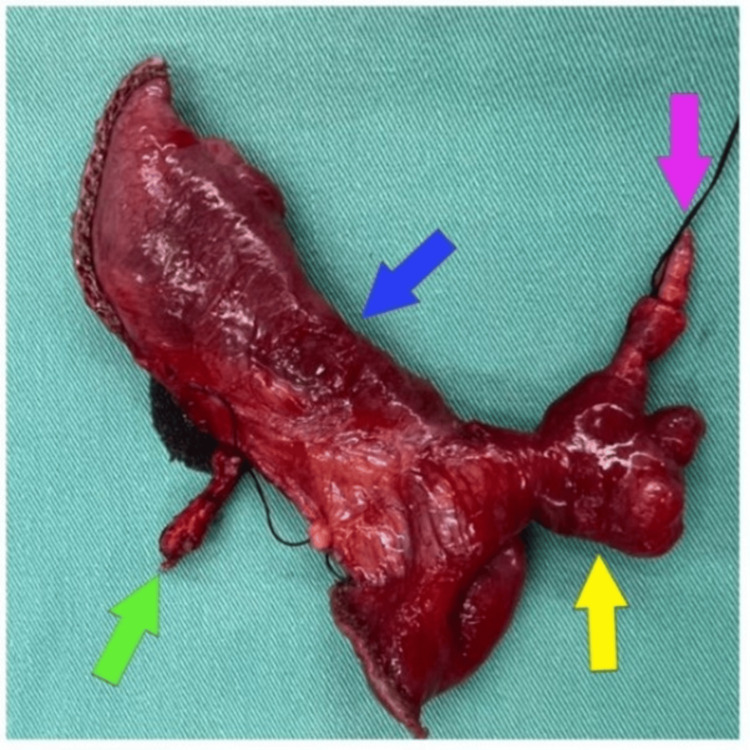
Resected small bowel specimen showing meso-diverticular band (green arrow), resected small bowel (blue arrow), Meckel's diverticulum (yellow arrow), and the other end of the meso-diverticular band (purple arrow).

Although the small bowel was viable with no evidence of gangrene or perforation, we proceeded with segmental resection involving only the portion of small bowel bearing the MD and its associated MDB (Figure 4).

**Figure 5:**
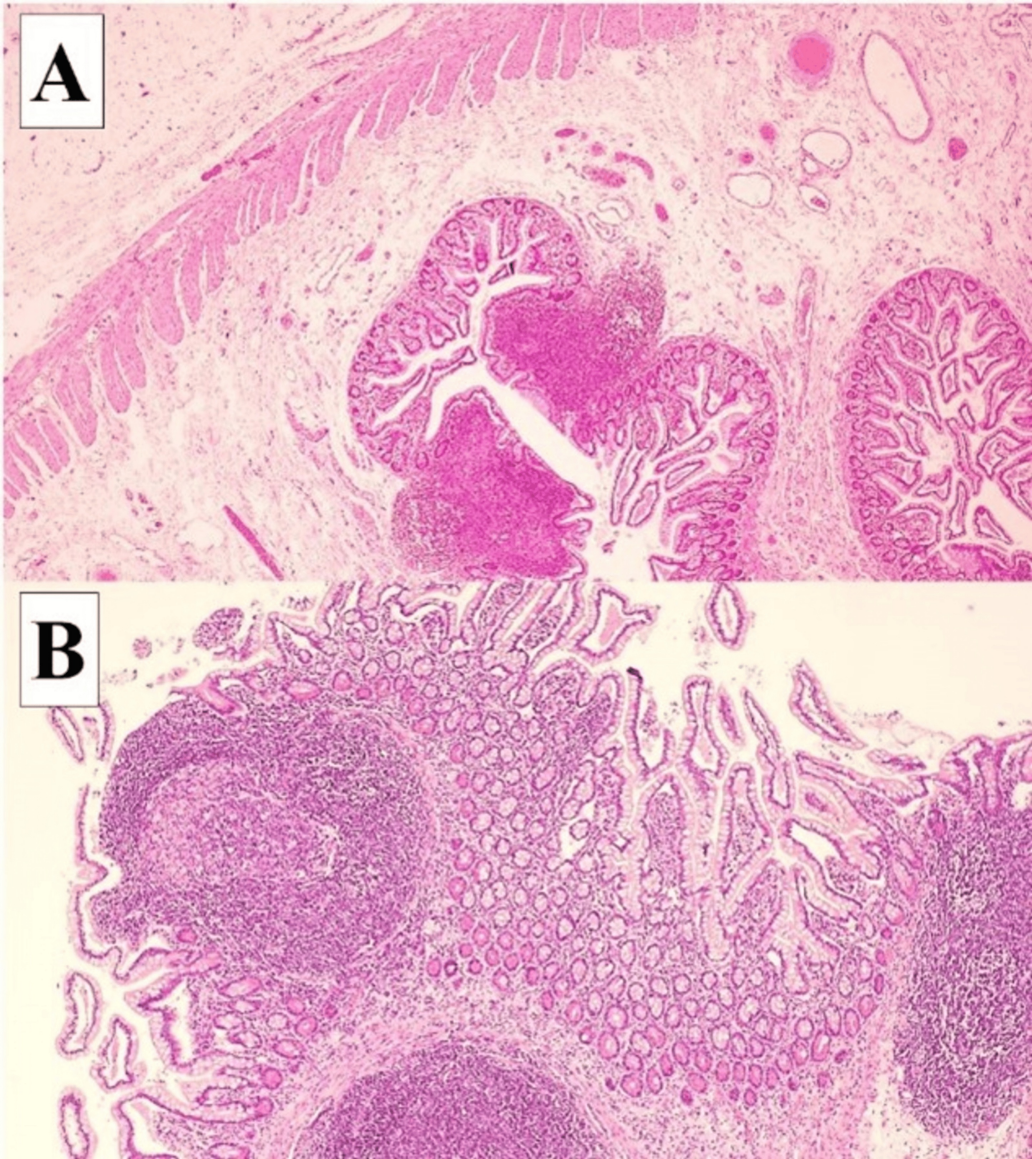
Histopathology image of specimen showing the following: A) The diverticulum comprised all layers of the small bowel, mucosa, submucosa, and muscular layer (H&E, x4). B) There are many reactive lymphoid follicles with prominent germinal center in the mucosa, with neither gastric nor pancreatic heterotopia identified (H&E, x10).

This decision was made because the MD and MDB constituted the clear anatomical lead points for the volvulus, and their removal was necessary to eliminate the risk of recurrent volvulus or future obstruction. The segment involved was limited, and resection with primary anastomosis was performed safely without compromising bowel length or function. The histopathology examination (HPE) was consistent with MD described as a blind-ended pouch containing all layers of small bowel wall, with no dysplasia or heterotopic gastric or pancreatic tissue seen. Both resected margins were viable, and no evidence of perforation, granuloma, or malignancy was seen (Figure 5).

Postoperatively, the patient recovered well with no complications, was gradually able to tolerate oral intake, and her gastrointestinal tract returned to normal function, and she was discharged home five days later. At her most recent follow-up, approximately one-and-a-half years postoperatively, the patient remained clinically well. Oral intake was satisfactory, and physical examination revealed a soft, non-tender abdomen with a well-healed surgical scar showing mild keloid formation. She described only occasional mild discomfort but had no significant functional limitations. Overall, the patient demonstrated a good recovery trajectory and expressed satisfaction with her postoperative outcome and quality of life.

## Discussion

This case is unique in several respects. First, it involves a young adult female with SBV secondary to an MD located on the mesenteric border of the ileum. This is a rare anatomical variant that diverges from the typical antimesenteric location. Additionally, the volvulus was caused by a persistent MDB that was firmly adherent to the anterior abdominal wall, resulting in a closed-loop obstruction, which is exceedingly rare and seldom documented. Most previously reported MDB-related obstructions involve internal herniation rather than volvulus anchored to the anterior wall. Furthermore, our decision to resect a segment of otherwise viable bowel highlights a preventive surgical strategy focused on eliminating the anatomical fulcrum for volvulus to avoid recurrence. These combined features underscore the value of this case in informing diagnostic suspicion and operative decision-making for obscure causes of bowel obstruction in young adults.

SBV can be classified based on its etiology into primary and secondary SBV. Primary SBV happens without anatomically abnormal predisposing factors, whereas secondary SBV is when the torsion is caused by an abnormal underlying pathology, such as adhesion bands, tumors, and MD [2,5]. Common symptoms of SBV at presentation are similar to an obstructed bowel, which include abdominal pain, distension, vomiting, and constipation [2]. In more serious circumstances, they may present acutely with bowel ischemia and/or peritonitis.

MD is a remnant of the vitello intestinal duct (also known as the omphalomesenteric duct). It arises from an incomplete obliteration of the embryonic vitelline duct, which connects the fetal gut to the yolk sac, typically regressing between the fifth and seventh weeks of gestation [6]. MD occurs in about 2% of people, with a 4-6% lifetime risk of becoming symptomatic [7]. The most common symptoms of MD in the adult population are obstruction, hemorrhage, and inflammation, while in the pediatric population, it is a painless rectal bleed. MD, when present, is located at the antimesenteric margin of the ileum with a mean distance of 52.4 cm (range 7-200 cm from the ileocecal valve) [8]. The intestinal mucosa lining the walls of the ileum also lines the walls of the MD, but at times, they may contain ectopic tissues. A systematic review showed the presence of ectopic tissues in 4.6-71.0% of all symptomatic MD. The most common ectopic tissues are of gastric and pancreatic tissues that comprise up to 97% of all ectopic tissues studied [8]. In our patient, the HPE shows the walls of MD composed of the small bowel lining with no ectopic tissues identified (Figure 5). Although not entirely accurate, the “rule of 2s” is commonly cited in clinical teaching to recall the features of MD, where it occurs in approximately 2% of the population, located around 2 feet from the ileocecal valve, and measuring roughly 2 inches in length. While this heuristic offers a simplified overview, our case highlights the importance of considering anatomical variants, such as a mesenteric-side Meckel’s, which fall outside this traditional framework and may contribute to atypical presentations like volvulus [8]. At times, it is accompanied by MDB, which is the congenital remnant of the vitelline artery. MDB increases the frequency of complications in the form of internal herniation or small intestine volvulus [6].

Investigating the cause of SBV may present a real challenge. Plain abdominal X-ray may show small bowel dilatation in general, but is not helpful in identifying SBV and the underlying cause. A CECT scan may guide in giving vital information, such as the “whirl sign” and “barber’s pole sign,” which are indicative of torsion [5]. In our patient, the CT scan showed SBV; however, there was no obvious etiology identified. MD, when observed in CECT, takes the shape of a cyst or blind pouch diverging from the ileum. It can be extremely difficult to discern MD from the adjacent loops in the small intestine in SBV [8]. Besides these imaging techniques, three-dimensional angiography may be valuable for diagnosis since it reveals interrupted blood flow in the SMV due to torsion [5]. In the literature, nuclear scans with Tc-99m pertechnetate may visualize the MD, taking advantage of the way the tracer accumulates in certain tissues like ectopic gastric tissue, which may be found in the MD. From a systematic review, the Tc-99m pertechnetate nuclear scan had a sensitivity of 89.6% and a specificity of 97.1% based on 562 scans that were done [8]. However, several factors can influence the diagnostic accuracy of the Tc-99m pertechnetate scan. The test relies on the presence of functional ectopic gastric mucosa within the MD, as the radiotracer is selectively taken up by gastric mucosa. Therefore, true positive results depend on the quantity and functionality of this ectopic tissue [8]. Conversely, false-negative results may occur if the ectopic mucosa is absent or insufficient, while false-positive results may arise from tracer accumulation in adjacent organs such as the stomach, kidneys, or urinary bladder, which can obscure or mimic uptake in the diverticulum [8]. Additionally, active gastrointestinal bleeding may cause extravasation of the tracer, further complicating image interpretation and potentially contributing to diagnostic inaccuracy [8].

Surgery is the mainstay of management for SBV. Urgent surgery is needed to prevent small bowel ischemia and peritonitis. Laparoscopy serves as both a diagnostic and therapeutic tool in the management of MD. Recent literature highlights the importance of early surgical intervention, typically involving diverticulectomy and, when necessary, bowel resection with anastomosis [6]. A literature review done on MD- and MDB-related small bowel obstruction reported 16 cases published in the last five years, which shows that all patients were treated with surgery [6]. A total of 75% of them underwent laparotomy, and the remaining 25% underwent laparoscopy. Of the patients who underwent laparoscopic surgery, 50% of them underwent small bowel resection for ischemic bowel [6]. One patient post laparotomy had postoperative fever and intra-abdominal collection, and there were no complications post laparoscopic bowel resection. Laparoscopy also provides better outcomes and, on average, a shorter postoperative stay compared to laparotomy cases [6]. Interestingly, 58% of patients who underwent exploratory laparotomy had bowel ischemia needing resection.

In another literature review, laparotomy was the surgical approach of choice, with six out of eight patients undergoing laparotomy [5]. Seven patients (87.5%) underwent devolvulation of SBV while one underwent bowel resection and enteropexy. Devolvulation alone has been reported to carry a recurrence rate of 30%. Given the high recurrence rate, there remains no clear consensus on whether bowel resection is necessary following a successful devolvulation [5]. While some consider enteropexy an effective measure for preventing recurrence, there have been reported cases of fistula formation postoperatively [5].

In a retrospective study done in the United States over 13 years (1998-2010), published in 2015, there were 2,065,599 hospitalizations for bowel obstruction, out of which 20,680 hospitalizations (1.00%) were attributed to SBV. The major strength of this study was the large sample size. A total of 13,486 (65.21%) patients underwent surgery, while the remaining 7194 (34.79%) patients were treated with nonoperative management, which includes nasogastric decompression, supportive care, and mechanical ventilation [9]. The reason for conservative management was not disclosed. A total of 855 (6.34%) patients underwent laparoscopic surgery, while the rest underwent laparotomy. Notably, the mortality rate among patients managed nonoperatively was nearly double that of those who underwent surgical intervention (11.65% vs. 5.94%), underscoring the critical importance of timely surgical management [9]. In our case, early operative intervention not only confirmed the rare anatomical cause but also facilitated definitive treatment, leading to an excellent clinical outcome [9].

## Conclusions

SBV secondary to MD with an MDB is rare, particularly when the MD arises from the mesenteric border and the MDB is fixed to the anterior abdominal wall, creating a closed-loop obstruction. This atypical anatomy complicates preoperative diagnosis and reinforces the need for early surgical exploration. Resection of the MD and MDB is essential, even in the absence of bowel ischemia, to prevent recurrence. This case emphasizes the importance of recognizing anatomical variants of MD in the differential diagnosis of acute intestinal obstruction.
